# Polyborosilazanes with Controllable B/N Ratio for Si–B–C–N Ceramics

**DOI:** 10.3390/ma16031053

**Published:** 2023-01-25

**Authors:** Yanpei Dang, Tianhao Li, Yangzhong Zhao, Liantai Duan, Jianning Zhang, Ke Chen, Liu He, Qing Huang, Chuanzhuang Zhao, Yujie Song

**Affiliations:** 1 School of Materials Science and Chemical Engineering, Ningbo University, Ningbo 315211, China; 2 Engineering Laboratory of Advanced Energy Materials, Ningbo Institute of Materials Technology and Engineering, Chinese Academy of Sciences, Ningbo 315201, China; 3 Qianwan Institute of CNITECH, Ningbo 315336, China

**Keywords:** polyborosilazane, SiBCN, carborane, polymer derived ceramics, B/N ratio, microphase evolution

## Abstract

Polyborosilazanes with controllable B/N ratio were synthesized using high-boron-content *m*-carborane, dichloromethylsilane, and hexamethydisilazane. After high-temperature pyrolysis, Si–B–C–N quaternary ceramics with SiC and B_4_C as the main phases were obtained. The B/N ratio in the precursors corresponded to the change in the feeding ratio of carborane and dichloromethylsilane. The effects of boron content and B/N ratio on the ceramic precursors and microphase structure in Si–B–C–N quaternary ceramics were explored in detail through a series of analytical characterization methods. A high boron content results in a significant increase in the ceramic yield (up to 71 wt%) of polyborosilazanes, and at the same time, the B/N molar ratio was regulated from 28.4:1 to 1.62:1. The appearance of the B_4_C structure in the Si–B–C–N quaternary ceramics through the regulation of the B/N ratio, has rarely been reported.

## 1. Introduction

In the past decades, the preparation of quaternary Si–B–C–N ceramics, which were composed of silicon, boron, carbon, and nitrogen, through the polymer-derived ceramics (PDC) route, has been extensively investigated. Ceramics prepared via the PDC route have many advantages over those prepared via traditional powder technology, such as high purity, controllable molecular structures, and uniform element distribution [1]. The incorporation of boron atoms into the ceramics network endows Si–B–C–N ceramics with better thermal stability, chemical stability, and creeping resistance at high temperatures than those of the SiC and Si_3_N_4_ ceramics [2]. Since boron inhibits the formation of Si_3_N_4_, thereby improving the high-temperature resistance of the ceramics, the regulation of the boron content in the ceramic skeleton is of great significance.

Several methods exist for introducing boron into a polymer network, and they can be categorized into the following two groups. Firstly, boron has been introduced into polyborosilazanes typically through the aminolysis polymerization of boron-containing monomers possessing Si–Cl or B–X bonds (with X=Cl or Br) in the presence of NH_3_ or polysilazane monomers. Secondly, polyborosilazanes have been usually prepared by reacting a Si-based polymer containing unsaturated groups, such as vinyl groups, with borane containing reactive sites [3,4]. To the best of our knowledge, many previous studies have adopted the mentioned above methods to adjust the feeding number of boron-containing compounds, with the aim of adjusting the ratio of silicon and boron elements in the ceramic precursors and exploring the effect of this ratio on the structure of the polymers and ceramics [5]. Riedel and his coworkers found that Si–B–C–N ceramics were stable up to 2000 °C when the Si/B molar ratio was 1:3 [6]. However, many studies that investigated the regulation of the boron content have focused on a low boron content range, which could not accurately explain the effect of boron content on the structure and properties of Si-based ceramics [7,8]. Furthermore, most of the existing Si–B–C–N quaternary ceramic systems were composed of structures such as SiC, Si_3_N_4_, or BN, but the B_4_C structure has rarely been investigated. B_4_C has high thermal stability, a large neutron absorption cross-section, and excellent mechanical and thermoelectric properties, which render this compound a *p*-type semiconductor even at very high temperatures [9,10,11]. Therefore, the appearance of the B_4_C phase in the Si–B–C–N ceramic skeleton may considerably expand the application prospect of the ceramics.

Carborane (C_2_B_10_H_12_), which contains ten boron atoms and two carbon atoms in a molecule, is an icosahedral cluster compound. This chemical structure endows carborane with a high boron content and excellent chemical and thermal stability [12]. It has three isomers namely 1,2-carborane (*o*-carborane), 1,7-carborane (*m*-carborane) and 1,12-carborane (*p*-carborane). In this study, *m*-carborane was used as the boron source to react with dichloromethylsilane (DCMS) and hexamethyldisilazane (HMDS) to synthesize polyborosilazanes. In order to explore the effect of different B/N ratio on the high-temperature microphase structure of Si–B–C–N quaternary ceramics, the boron content in the polymers were adjusted by controlling the feeding ratio of carborane and DCMS to obtain different B/N ratio in the final products.

## 2. Experimental Section

### 2.1. Materials

All reactions were conducted under purified Ar using Schlenk techniques. *m*-Carborane was purchased from Zhengzhou Yuanli Technology and dried before use. *n*-Butyllithium was obtained from Aladdin Biochemical Technology Co., Ltd. (Shanghai, China) and dissolved in hexane at a concentration of 2.5 M. DCMS and HMDS were purchased from J&K Scientific Co. (San Jose, CA, USA) and were distilled before use. Solvents used: tetrahydrofuran, *n*-hexane, and xylene were also purchased from J&K Scientific Co. (water ≤ 50 ppm, with molecular sieves). The as-prepared precursors were stored in an Ar glove box until pyrolysis or analysis. 

### 2.2. Characterization

#### 2.2.1. Elemental Analysis

The oxygen and nitrogen contents of the precursors and the Si–B–N–C ceramics were determined by using an oxygen/nitrogen analyzer ON836 (LECO Co., San Jose, CA, USA), while the carbon content was determined using a carbon/sulfur analyzer CS844 (LECO Co., San Jose, CA, USA). The silicon and boron contents in the samples were determined utilizing inductively coupled plasma optical emission spectroscopy (ICP-OES, SPECTRO Co., Kleve, Germany). The connecting ways of chemical bonds of ceramics were characterized by X-ray photoelectron spectroscopy (XPS, Thermo Fisher K-Alpha+, Waltham, MA, USA) with Al Kα radiation.

#### 2.2.2. Structure Analysis and Microphase Evolution

Fourier-transform infrared (FTIR) spectra were collected using an Invenio R (Bruker Co., Saarbrucken, Germany) with KBr pellets, and the spectra were collected in the range of 400–4000 cm^−1^. The ^1^H NMR, ^11^B NMR, and ^13^C NMR of polyborosilazanes were tested on Bruker Avance 400M (Bruker Co., Saarbrucken, Germany). Samples were dissolved in CDCl_3_, and tetramethylsilane (TMS) was used as the reference for ^1^H NMR and ^13^C NMR. BF_3_OEt_2_ was used for ^11^B NMR as a reference. X-ray diffraction (XRD) studies were carried out using a D8 Advance Davinci (Bruker Co., Saarbrucken, Germany) with Cu Kα radiation (k = 1.54178 A) in the angular range 2*θ* = 5° to 90° at room temperature.

#### 2.2.3. Study on Polymer-to-Ceramic Transformation

The polymer-to-ceramic transformation of precursors was investigated by means of thermogravimetric analysis/mass spectroscopy (TGA/MS) using a TGA 8000-Spectrum two-Clarus SQ8T (PerkinElmer Co., Waltham, MA, USA). 

#### 2.2.4. Morphology

Transmission electron microscopy (TEM) Tecnai F20 (FEI Co., Hillsboro, OR, USA) was used for collecting microstructure pictures of ceramics. Ceramics were ground to less than 100 nm, ultrasonic dispersed in ethyl alcohol, a blob of the solution was dropped on a 3 mm carbon-coated copper grid, and the accelerating voltage was set as 200 kV for imaging. The morphology of ceramic bulk was examined with the scanning electron microscope (SEM) S-4800 (HITACHI Co., Tokyo, Japan) equipped with EDS from Oxford Co., Oxford, UK.

### 2.3. Synthesis of the Polymers 

#### 2.3.1. Synthesis of the Monomer CB

**Scheme 1:**
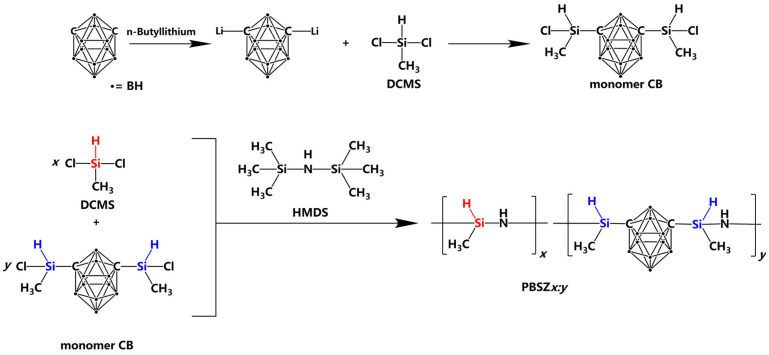
Synthesis pathway of PBSZ*x:y*.

The synthesis of the monomer was carried out in a 250 mL Schlenk reactor with a magnetic stirrer and an Ar inlet. Firstly, 3.76 g of *m*-carborane (0.025 mol) was dissolved in 40 mL of tetrahydrofuran (THF) and cooled to approximately 0 °C. Under vigorous stirring, 20 mL (0.05 mol) of *n*-butyllithium was added to this solution. As this reaction is a highly exothermic neutralization process, the temperature should be held at 0 °C. After allowing the mixture to warm to room temperature, it was stirred for 4 h. This mixture was then added to 10 mL (0.10 mol) of DCMS at 0 °C. Next, the mixture was warmed up to room temperature and stirred for 18 h. Subsequently, the by-products of the reaction were filtered, and all volatile components were removed in a vacuum to produce a faint yellow and highly viscous liquid named monomer CB, which was very sensitive to moisture and air (Scheme 1). 

#### 2.3.2. Synthesis of Polymers 

The synthesis of the polymers was carried out in a 250 mL Schlenk reactor with a mechanical stirrer, a thermocouple, and an argon inlet. Firstly, 8 mL (0.075 mol) of DCMS, 0.025 mol monomer CB, and 40 mL of xylene were added to the flask at 0 °C. Next, 80 mL (0.38 mol) of HMDS was added to this mixture. Then, *n*-hexane and the by-products were distilled off by slowly heating the mixture up to 150 °C to yield the product as a viscous liquid. Finally, this viscous liquid was subjected to a subsequent thermal treatment up to a temperature of 280 °C, which increased the viscosity of the polymer due to the increase of the cross-linking degree in the system (caused by polycondensation). The polymeric product at this high temperature formed a highly viscous mass, which solidified into brittle chunks at ambient temperature, labeled PBSZ3:1. The other samples were synthesized using the same method but with different molar ratios of DCMS to monomer CB, and the samples were correspondingly named PBSZ*x:y* (where *x:y* is the molar ratio of DCMS and monomer CB).

### 2.4. Pyrolysis of the Polymers

When the pyrolysis temperature was below 1000 °C, the samples were placed in an alumina boat and heated in a quartz tube furnace under N_2_ flow. Because N_2_ or other gases will react with Si-B-C-N ceramics above 1000 °C [13], when the pyrolysis temperature was above 1000 °C, the samples were placed in a graphite crucible and heated in a graphite furnace under Ar flow. During the pyrolysis process, the temperature progressively increased up to 200 °C at a rate of 5 °C/min. After holding the temperature at 200 °C for 1 h, the temperature increased again (heating rate: 5 °C/min below 1000 °C and 10 °C/min above 1000 °C) until the pyrolysis temperature was reached. The samples were kept at the pyrolysis temperature for 1 h and then were cooled to ambient temperature. After pyrolysis, these polymer-derived ceramics were named SiBCN*x:y* (where *x:y* is the molar ratio of DCMS and monomer CB).

## 3. Results and Discussion

### 3.1. Polymer Synthesis and Characterization

Due to the good properties of *m*-carborane, such as their relatively high boron content and their excellent thermal and chemical stability, we developed a novel method for the synthesis of polyborosilizanes with different B/N ratios based on *m*-carborane, HMDS, and DCMS. In this process, the B/N ratio in polyborosilazanes was adjusted by changing the feeding ratio of DCMS and monomer CB. We designed polymers with controlled B/N ratios to study the chemistry behind the synthesis steps of these polymers and understand the effect of different B/N ratios on the microphase evolution of the Si–B–C–N ceramics at high temperatures. PBSZ*x:y* can be either brittle solids (PBSZ0:1 and PBSZ3:1) or viscous solids (PBSZ6:1 and PBSZ8:1) depending on the quantity of *m*-carborane that presents in the polymer structure. These precursors are soluble in common organic solvents (chloroform, tetrahydrofuran, etc.) and have the right melting temperature. Thus, they can be processed easily. The preparation route of the ceramic precursors based on *m*-carborane is shown in Scheme 1.

**Figure 1:**
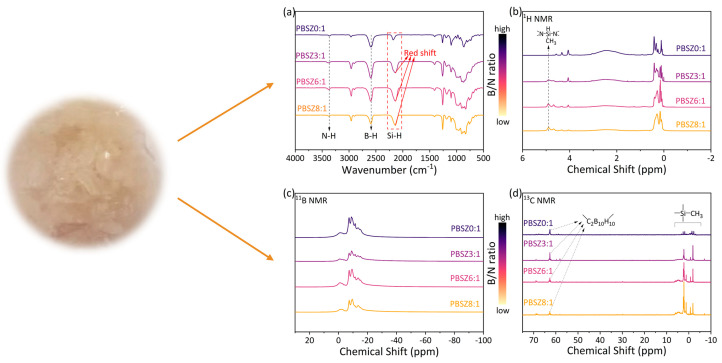
(**a**) FTIR spectra recorded for PBSZ*x:y*, (**b**) ^1^H NMR, (**c**) ^11^B NMR, and (**d**) ^13^C NMR recorded for PBSZ*x:y*.

The FTIR spectra shown in Figure 1a reveal that the structural units in the investigated polymers are similar. All the peak positions are nearly the same across the spectra, indicating virtually identical chemical structures for the four polymers. The asymmetric stretching vibrations of the N–H and Si–H units are observed at around 3380 and 2177 cm^−1^, respectively, but the Si–H bonds undergo a clear redshift as the value of *x:y* increase [14]. From the FTIR spectra, except for the case of PBSZ0:1, the Si–H groups can be observed for different chemical environments (Scheme 1), which is caused by the different polymer structures in turn due to the different ratios of DCMS and monomer CB. This phenomenon is more pronounced for PBSZ3:1. The sharp peaks at 2960 cm^−1^ are attributed to the *ν*(C–H) bonds, the peaks at 1405 cm^−1^ are attributed to *δ*(C–H) bonds, and the symmetric deformation vibration of the Si–CH_3_ bonds at 1260 cm^−1^ originate from either DCMS or HMDZ. Furthermore, the B–H and B–C peaks at 2590 and 1189 cm^−1^, respectively, confirm the existence of the *m*-carborane structures [15]. Finally, the signals of Si–N–Si structures are observed at 945 cm^−1^ in all polymers [16]. Despite their similar structures, an essential difference appears among the four precursors, namely the fact that the area ratios of the B–H and N–H peaks decrease gradually with increasing the *x:y* value. This truth illustrates that the ratios of the B–H bonds to N–H bonds decrease as a larger amount of DCMS is introduced.

The NMR spectroscopy analysis was performed for monomer CB and all polymer samples. The features of the ^1^H, ^11^B, and ^13^C NMR spectra of the different samples are discussed below to understand the specific structure of these samples.

From Figure S1a and Figure 1b, the ^1^H signals are detected at *δ* = 0.01–0.76(Si–CH_3_), 1.13–3.63(B–H, from *m*-carborane), and 3.95–4.90 ppm (Si–H), which are similar for all samples. However, there are several small differences. Firstly, compared with monomer CB, the Si–CH_3_ and Si–H peaks located at *δ* = 0.61 and 4.79 ppm, respectively, disappear in all polymers, indicating that monomer CB and/or DCMS have been polymerized with HDMS, resulting in the disappearance of the –CH_3_SiH–Cl structure. (Scheme 1) Secondly, for PBSZ3:1, PBSZ6:1, and PBSZ8:1, the appearance of the peaks at *δ* = 4.90 ppm is due to the formation of the N–(CH_3_)SiH–N structure, whereas this structure is not formed in monomer CB and PBSZ0:1. This is caused by the reaction of DCMS and HDMS in PBSZ3:1, PBSZ6:1, and PBSZ8:1 systems. In addition, the peaks at *δ* = 4.33 represent the Si–H bonds in the chemical environment of –(C_2_B_10_H_10_)–SiH–N–, which can be observed in all polymers. The above two Si–H structures are in good agreement with the FTIR spectra. Finally, we can observe that with the introduction of DCMS, the B–H peaks (*δ* = 1.13 to 3.63 ppm) in the ^1^H NMR spectra become gradually less intense, which demonstrates that with increasing ratio of DCMS and monomer CB, the boron content decreases significantly (that is, the B/N ratio is adjusted). In order to further probe the structure of these polymers, ^11^B NMR and ^13^C NMR spectroscopy analyses were carried out on these samples, which clearly show the chemical environments of boron and carbon within these polymers. From Figure S1b and Figure 1c, boron in different chemical environments in the carborane cage can be clearly observed, including the B–B, B–C, and B–H bonds (*δ* = −15–0 ppm), and the spectra contain characteristic broad lines, which are due to the unresolved ^11^B–^11^B and ^11^B–^10^B couplings [17]. Additionally, the chemical environment of carbon can be observed from Figure 1d; the –Si–C_2_B_10_H_10_–Si– and –Si–CH_3_ are located at around *δ* = 60 and −3–3 ppm, respectively [18]. Furthermore, it can be observed that in the range from PBSZ0:1 to 8:1, the peak of the area ratios of the –Si–C_2_B_10_H_10_–Si– and –Si–CH_3_ structures gradually decrease (1:2 for PBSZ0:1, 1:6 for PBSZ3:1, 1:11 for PBSZ6:1 and 1:22 for PBSZ8:1), which demonstrates the reduction of the carborane cage structure in the Si-based polymers; that is, the polymer structure is regulated.

The FTIR and NMR results demonstrate that the carborane cage structure was successfully introduced into the polymer system. In addition, upon changing the feeding ratio of DCMS and monomer CB, the B/N ratio in the Si-based polymers is adjusted.

### 3.2. Polymer-to-Ceramic Conversion

**Figure 2:**
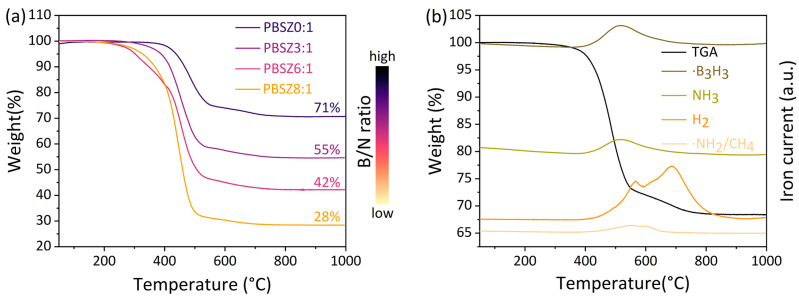
(**a**) TGA curves of PBSZ*x:y* pyrolyzed under Ar atmosphere and (**b**) TGA-MS curves of PBSZ0:1 pyrolyzed under Ar atmosphere.

**Scheme 2:**
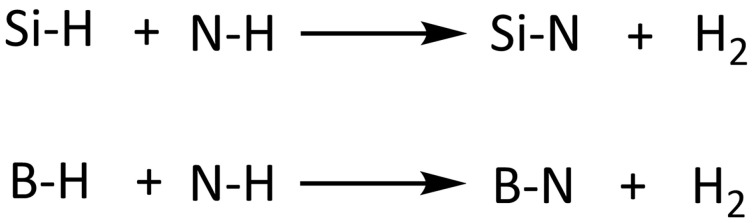
Cross-linking reaction of Si–H and B–H with N–H.

The thermal conversion of preceramic polymers into ceramics is accompanied by the formation of gaseous products from the polymers; therefore, weight loss occurs. Here, we investigated the polymer-to-ceramic conversion of different polymer samples under argon flow from 50 °C to 1000 °C via TGA (Figure 2a). Between 250 °C and 500 °C, a significant weight loss can be observed, which is mainly due to the generation of oligomers with low molecular weight and the occurrence of cross-linking processes in the systems. In this stage, although the weight loss is similar across all the polymers, there is a small difference. We can see that with increasing *x:y* value, that is, with decreasing carborane amount in these polymer backbones, the initial weight loss temperature of the polymers is reduced from 400 °C (PBSZ0:1) to 250°C (PBSZ8:1). The weight loss rate in the temperature range of 500–800 °C is lower than that in the previous stage, which is due to the ceramization process of the ceramic precursors to yield Si–B–C–N quaternary ceramics. This process is accompanied by the fragmentation of the cage carborane structure and the formation and rearrangement of free radicals. From the TGA/MS data of PBSZ0:1 (Figure 2b), it can be seen that H_2_ and NH_3_ and ·B_3_H_3_ and ·NH_2_/CH_4_ are generated. The signals of these substances appear at temperatures exceeding 400 °C; in particular, the signals of NH_3_ and ·B_3_H_3_ peak at 500 °C, while the signal of ·NH_2_/CH_4_ peaks at 600 °C. However, these signals gradually weaken as the temperature increases further, and they disappear completely above 800 °C. H_2_ shows two peaks, a weak peak around 550 °C and a strong peak around 700 °C, and the H_2_ signal gradually weakens at higher temperatures until it disappears above 800 °C (Scheme 2). The two hydrogen peaks may originate from the dehydrogenation reaction of the Si–H and N–H bonds and the reaction of the N–H and B–H bonds from the fragmentation of the carborane cages. This information proves that the ceramization transition process ends at a temperature below 800 °C as the ceramics no longer undergo a mass change at higher temperatures. It is worth mentioning that the thermal decomposition of the preceramic probably occurs through mechanisms that overlap with each other rather than being well separated in clear temperature ranges. Finally, the ceramic yields of PBSZ0:1, PBSZ3:1, PBSZ6:1, and PBSZ8:1 are 71 wt%, 55 wt%, 42 wt%, and 28 wt%, respectively. This is due to the resonance effect of the carborane structure that stabilizes the polymer structures. Thus, the ceramic yield decreases as the number of carboranes in the polymer structures decreases.

The elemental analysis of the ceramics treated at 1000 °C is shown in Table 1. As mentioned above, the precursors have completed the ceramic transformation process and almost have no hydrogen residue at 1000 °C. Therefore, polymer-derived ceramics consist of silicon, boron, carbon, nitrogen, and oxygen. The data show that the B/N ratio in the Si–B–C–N ceramics can be effectively regulated by changing the feeding ratio, which is consistent with the FTIR and NMR results presented above. Additionally, the calculated compositions of SiBCN0:1, SiBCN3:1, SiBCN6:1, and SiBCN8:1 are Si_5.45_B_28.4_C_8.13_N, Si_2.07_B_4.62_C_2.44_N, Si_1.77_B_2.96_C_1.99_N, and Si_1.82_B_1.62_C_1.71_N, respectively.

### 3.3. Structural Evolution of the Polymer-Derived Ceramics

**Figure 3:**
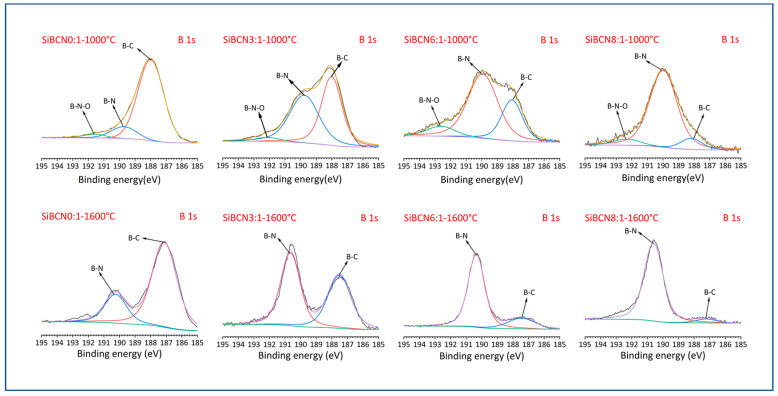
XPS spectra of B 1s of SiBCN*x:y* at 1000 °C and 1600 °C.

In order to clearly explore the structure of the ceramics, the XPS analysis of the different ceramics was carried out, suggesting the existence of Si, B, C, N, and O (Figure S2). From the Si2p spectra of the four ceramics, three peaks can be clearly observed, namely 100.1, 101.5, and 103.4 eV, which can be attributed to the Si–C, Si–N, and Si–O bonds. The B1s spectra show three distinct peaks, at 188.0 eV (B–C peaks), 189.8 eV (B–N peaks), and 192.1 eV (B–N–O peaks). Notably, the B–C peak areas reduce from the SiBCN0:1 to the SiBCN8:1, while the B–N peak areas increase. This suggests that as the B/N ratio decreases, the B–N bonds become the dominant form of boron in ceramic systems. This may be due to the lower nitrogen content for samples with a high B/N ratio, resulting in a lower proportion of the B–N structures in ceramic samples with a high B/N ratio. The C–C and C–B signals overlap at 284.80 eV, while the C–B–N signals are observed at 286.4 eV. In addition, the C–Si (282.4 eV) and C–O peaks also appear in the C1s spectra. The N–O (401.7 eV), N–B (398.2 eV), and N–Si (397.6 eV) peaks can be observed in the N1s spectra, which agrees with the results of the Si2p and B1s spectra. Additionally, it can be found that with the increase in the pyrolysis temperature, the B–C bonds are gradually transformed into the more stable B–N bonds (Figure 3). At the same time, with the increase in temperature, the peak area of Si–N bonds gradually decreases, and at 1600 °C, the existing form of nitrogen elements are only B–N bonds. In summary, the structures of the Si–B–C–N quaternary ceramics with high boron content consist of the Si–C, Si–N, B–N, B–C, B–N–O, C–C, C–B–N, and N–O bonds [5,19,20,21,22].

**Figure 4:**
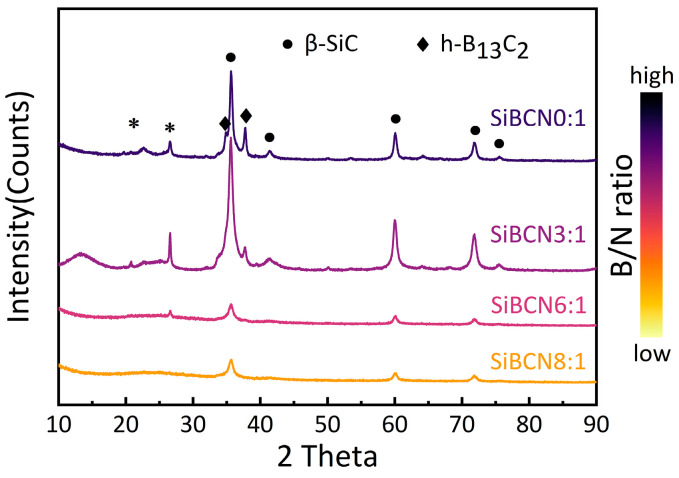
XRD spectra of SiBCN*x:y* annealed at 1600 °C in Ar gas (*: SiO_2_).

XRD measurements and TEM imaging were conducted to characterize the structure of the ceramic samples treated at 1600 °C for 1 h under an Ar atmosphere. The XRD patterns of SiBCN*x*:*y* annealed at 1600 °C are shown in Figure 4. The SiC peaks can be observed in all ceramic systems; in particular, the SiC peaks in SiBCN0:1 and SiBCN3:1 are sharper than those of the other samples. This is attributed to the fact that a high boron content promotes the crystallization of SiC [23]. Furthermore, the B_13_C_2_ (existence form of B_4_C with high boron content) peaks appear only in SiBCN0:1, SiBCN3:1, and SiBCN6:1 (a weaker signal). A possible reason for this behavior is that when SiC reaches a certain degree of crystallinity, the diffusion of silicon and carbon atoms becomes more difficult. However, there are more boron atoms in the ceramics with a higher boron content, which explains the appearance of the B_4_C crystal phase. However, SiBCN8:1 with a relatively low boron content may be due to the fewer B_4_C structures; thus, peaks corresponding to the B_4_C crystals are not observed in the XRD spectrum of SiBCN8:1, which is in line with the XPS results. With decreasing B/N ratio, the amount of the B–C bonds decreases while that of the B–N bonds increases, which leads to a decrease in the proportion of the B_4_C structure. The peaks corresponding to the SiO_2_ crystals can be observed in the XRD patterns of all samples except for SiBCN8:1, which is attributed to the inevitable introduction of oxygen during the experiment and the relatively high silicon content in these systems.

**Figure 5:**
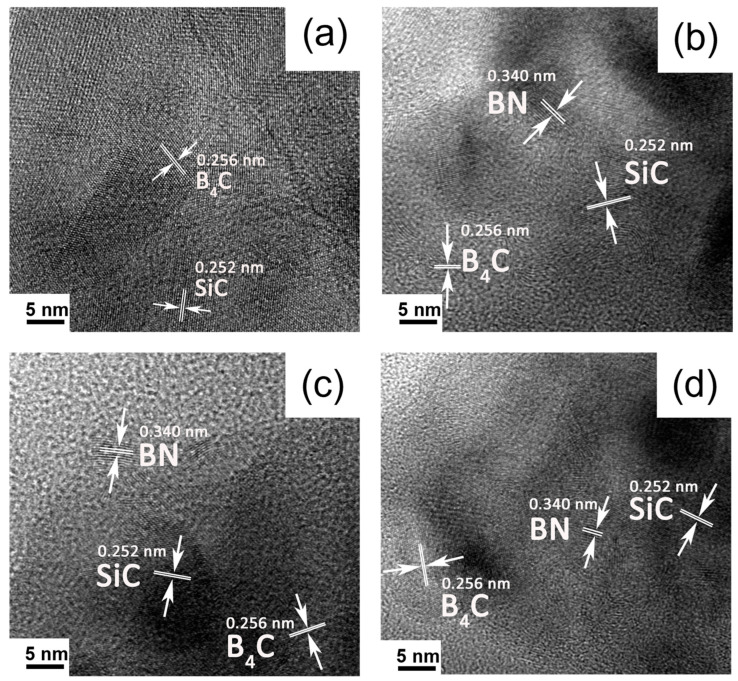
TEM images of SiBCN0:1 (**a**), SiBCN3:1 (**b**), SiBCN6:1 (**c**), and SiBCN8:1 (**d**) treated at 1600 °C.

These XRD results are confirmed by the TEM investigations (Figure 5), although several discrepancies also exist. The TEM image of SiBCN0:1 reveals the presence of lattice fringes with spacings of 0.256 and 0.252 nm, which correspond to the (104) lattice plane of B_4_C and the (111) lattice plane of SiC, respectively. For SiBCN3:1, SiBCN6:1, and SiBCN8:1, the (002) lattice plane of BN, the (111) lattice plane of SiC, and the (104) lattice plane of B_4_C correspond to the lattice fringe spacing of 0.340, 0.252 and 0.256 nm, respectively. Notably, the BN crystallites were detected by TEM in SiBCN3:1, SiBCN6:1, and SiBCN8:1 but were not detected by XRD. The possible reason for this discrepancy is that the higher nitrogen content in these systems than the SiBCN0:1 system results in an increased amount of BN structures. However, due to the excellent anti-crystallization property of BN, only a few nanocrystals are formed in these systems, which are not detected by XRD. 

Through the characterization of the Si–B–C–N quaternary ceramics, it is found that they are composed of BN, SiC, and B_4_C, among which SiC is the predominant structure. Furthermore, upon adjusting the B/N ratio, the microphase structures of the ceramics change accordingly. That is, the decrease in the B/N ratio leads to a decrease in the number of B–C bonds and an increase in the number of B–N bonds. Therefore, the B_4_C content in the ceramic systems can be adjusted by changing the B/N ratio.

### 3.4. Mechanical Properties of the Si–B–C–N Ceramics 

Since the main phases of the investigated ceramics are SiC and B_4_C, both of which have excellent mechanical properties (especially B_4_C), the mechanical properties of the ceramics were studied. As SiBCN0:1 has the highest B content, that is, it has the largest proportion of the B_4_C structures, SiBCN0:1 was selected as the raw material. The ceramic powers were heated to 1600 °C at a rate of 50 °C/min under a pressure of 50 MPa in an Ar atmosphere using spark plasma sintering (SPS) to prepare a ceramic bulk. The surface topography of the ceramic bulk is shown in Figure S3. Several defects can be observed, indicating that the bulk is not very dense, and the density of this bulk is 2.40 g/cm^3^. However, in this experiment, the sample exhibits excellent performance, with the hardness and elastic modulus reaching 26.2 and 337.3 GPa, respectively. The effect of the composition and structure of the precursors on the properties of the ceramic bulks will be reported in detail in future work.

## 4. Conclusions

In this present work, polyborosilazanes with different B/N ratios have been prepared using *m*-carborane, dichloromethylsilane (DCMS), and hexamethyldisilazane (HMDS) and then the Si-B-C-N quaternary ceramics with different B/N ratio were fabricated via the PDC route. Firstly, the introduction of the *m*-carborane structure in the polyborosilazane systems significantly increased the boron content of the Si–B–C–N quaternary ceramics, up to 50 wt%, which was rarely reported. The B/N molar ratio of the Si-based ceramics could be regulated from 28.4:1 to 1.62:1 by adjusting the feeding ratio of DCMS to carborane-containing monomer CB. Secondly, the ceramic yields of polyborosilazanes increased considerably (from 28 wt% to 71 wt%) with the increasing proportion of the *m*-carborane structure in the molecular framework, and the initial weight loss temperature of the polymers also increased from 250 °C for PBSZ8:1 to 400 °C for PBSZ0:1. This is due to the resonance effect of the *m*-carborane cage structure, which enhances the thermal stability of the polymers. Thirdly, through the adjustment of the B/N ratio in ceramics, the microphase structures of the ceramics changed accordingly. The content of B_4_C phase content in Si-B-C-N ceramics could be readily adjusted, which has been rarely reported. Finally, SiBCN0:1 with high content of B_4_C structures was sintered via SPS to obtain a ceramic bulk with excellent mechanical properties, whose hardness and elastic modulus reached 26.2 and 337.3 GPa, respectively. This strategy will provide new insight into the design of Si–B–C–N quaternary ceramics and widen the applications in high-temperature absorbing materials and ultrahigh-temperature composite ceramics.

This study plays an important role in the design of ultrahigh-temperature composite ceramics. However, the cost of carborane is expensive, and the functionalization of carborane is complex, which limits its practical applications. The carborane-based polyborosilizanes provide a new way to prepare Si-B-C-N ceramics with high boron content, which can rarely be achieved through other methods.

## Figures and Tables

**Table 1:** Elemental composition of the ceramics pyrolyzed at 1000 °C under N_2_ gas.

Ceramics ^※^	Si (wt%)	B (wt%)	C (wt%)	N (wt%)	B/N Molar Ratio	Formula
SiBCN0:1	24.4	50.0	20.5	2.2	28.4:1	Si_5.45_B_28.4_C_8.13_N
SiBCN3:1	37.7	33.0	19.0	9.1	4.62:1	Si_2.07_B_4.62_C_2.44_N
SiBCN6:1	41.2	27.0	19.8	11.6	2.96:1	Si_1.77_B_2.96_C_1.99_N
SiBCN8:1	47.9	16.8	19.3	13.1	1.62:1	Si_1.82_B_1.62_C_1.71_N

※ Oxygen was omitted.

## Data Availability

Not applicable.

## Supplementary Materials

The following are available online at https://www.mdpi.com/article/10.3390/ma16031053/s1, Figure S1: (a) 1H NMR, and (b) 11 B NMR recorded for monomer CB; Figure S2: XPS spectra of SiBCNx:y pyrolyzed under Ar atmosphere at 1000 °C (a), 1200 °C (b), 1400 °C (c), 1500 °C (d), 1600 °C (e); Figure S3: SEM image of the ceramic bulk.

## Abbreviations

Abbreviations	Items
FTIR	Fourier Transform Ioncyclotron Resonance
NMR	Nuclear Magnetic Resonance
TGA	Thermal Gravimetric Analyzer
MS	Mass Spectrometry
XPS	X-ray Photoelectron Spectroscopy
XRD	X-ray Diffraction
TEM	Transmission Electron Microscope
SEM	Scanning Electron Microscope
HMDS	Hexamethyldisilazane
DCMS	Dicholoromethlsilane
THF	Tetrahydrofuran
SPS	Spark Plasma Sintering
