# Supplementary material for: Polyborosilazanes with Controllable B/N Ratio for Si–B–C–N Ceramics
Source: Materials (Basel). 2023 Jan 25;16(3):1053. doi: 10.3390/ma16031053 (PMC9918987; doi:10.3390/ma16031053)
Supplement: Supplementary file 1 [file materials-16-01053-s001.zip › materials-2090493-supplementary.pdf]

# 1. Characterization of the monomer CB

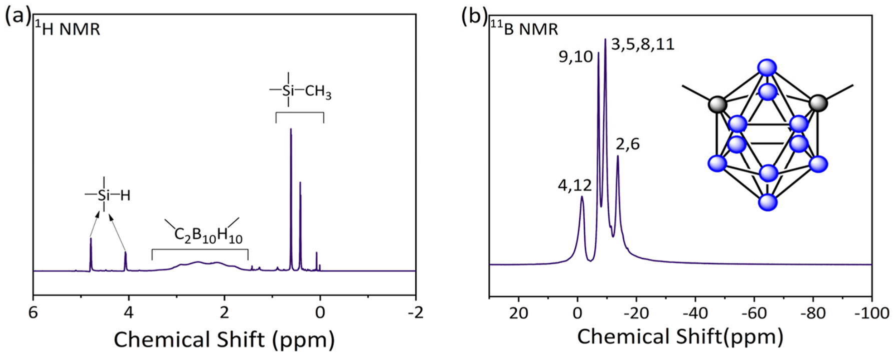


**Figure S1.** (**a**) $^{1}$H NMR, and (**b**) $^{11}$ B NMR recorded for monomer CB.

# 2. Structural evolution of the polymer-derived ceramics

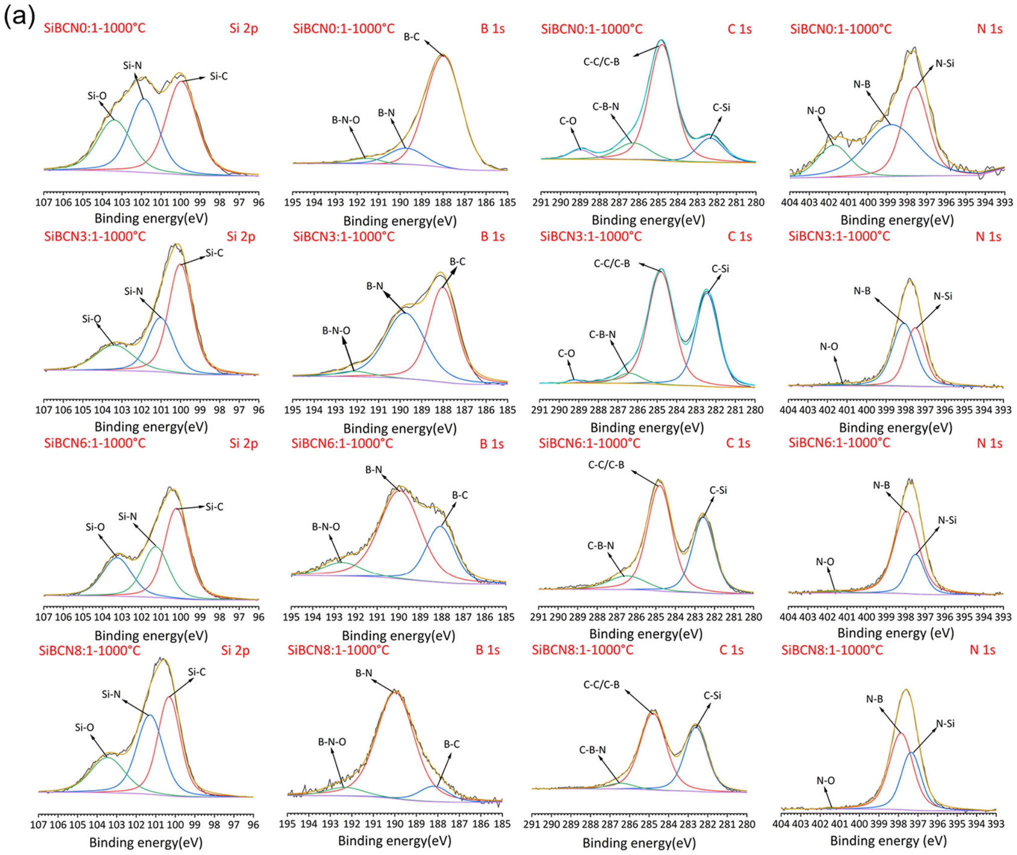

(b)

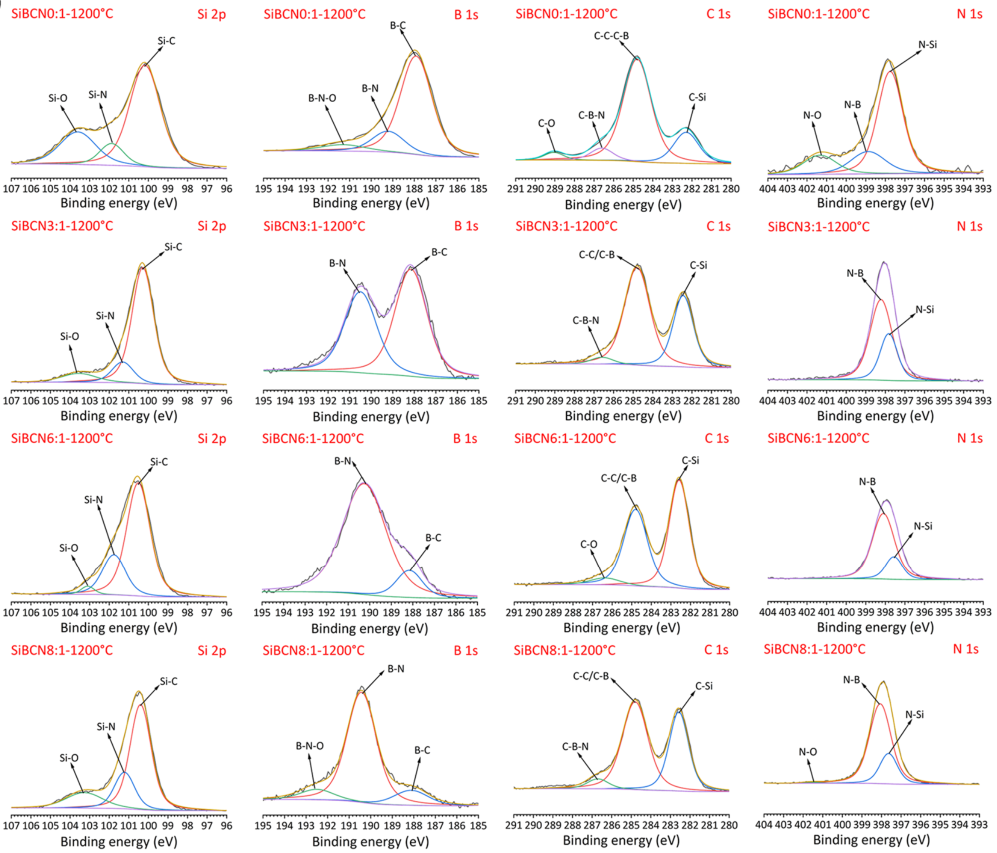

SiBCN0:1-1200°C
Si 2p
Si-C
Si-O
Si-N
B 1s
B-C
B-N-O
B-N
C 1s
C-C-C-B
C-O
C-B-N
C-Si
N 1s
N-Si
N-O
N-B
SiBCN3:1-1200°C
SiBCN6:1-1200°C
C-C/C-B
SiBCN8:1-1200°C
Binding energy (eV)

(c)

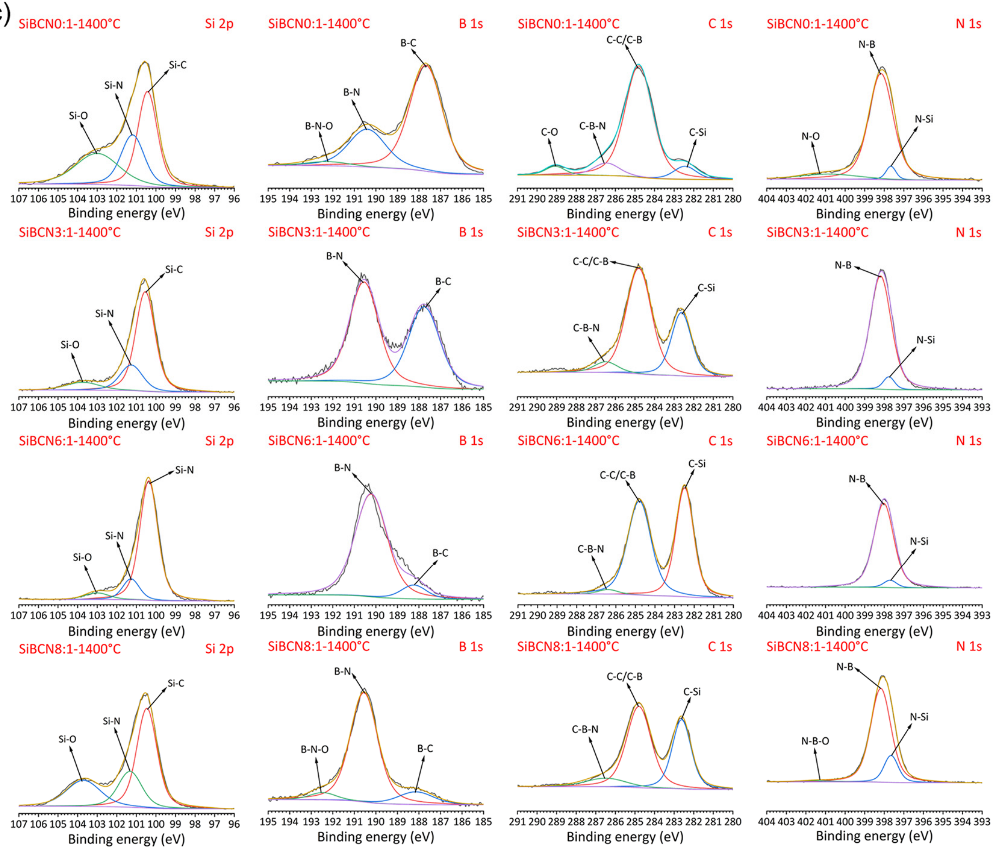
SiBCN0:1-1400°C
Si 2p
Si-O
Si-N
Si-C
Binding energy (eV)
SiBCN0:1-1400°C
B 1s
B-N-O
B-N
B-C
Binding energy (eV)
SiBCN0:1-1400°C
C 1s
C-O
C-B-N
C-C/C-B
C-Si
Binding energy (eV)
SiBCN0:1-1400°C
N 1s
N-O
N-B
N-Si
Binding energy (eV)
SiBCN3:1-1400°C
Si 2p
Si-O
Si-N
Si-C
Binding energy (eV)
SiBCN3:1-1400°C
B 1s
B-N
B-C
Binding energy (eV)
SiBCN3:1-1400°C
C 1s
C-C/C-B
C-B-N
C-Si
Binding energy (eV)
SiBCN3:1-1400°C
N 1s
N-B
N-Si
Binding energy (eV)
SiBCN6:1-1400°C
Si 2p
Si-O
Si-N
Si-N
Binding energy (eV)
SiBCN6:1-1400°C
B 1s
B-N
B-C
Binding energy (eV)
SiBCN6:1-1400°C
C 1s
C-C/C-B
C-B-N
C-Si
Binding energy (eV)
SiBCN6:1-1400°C
N 1s
N-B
N-Si
Binding energy (eV)
SiBCN8:1-1400°C
Si 2p
Si-O
Si-N
Si-C
Binding energy (eV)
SiBCN8:1-1400°C
B 1s
B-N
B-N-O
B-C
Binding energy (eV)
SiBCN8:1-1400°C
C 1s
C-C/C-B
C-B-N
C-Si
Binding energy (eV)
SiBCN8:1-1400°C
N 1s
N-B
N-B-O
N-Si
Binding energy (eV)

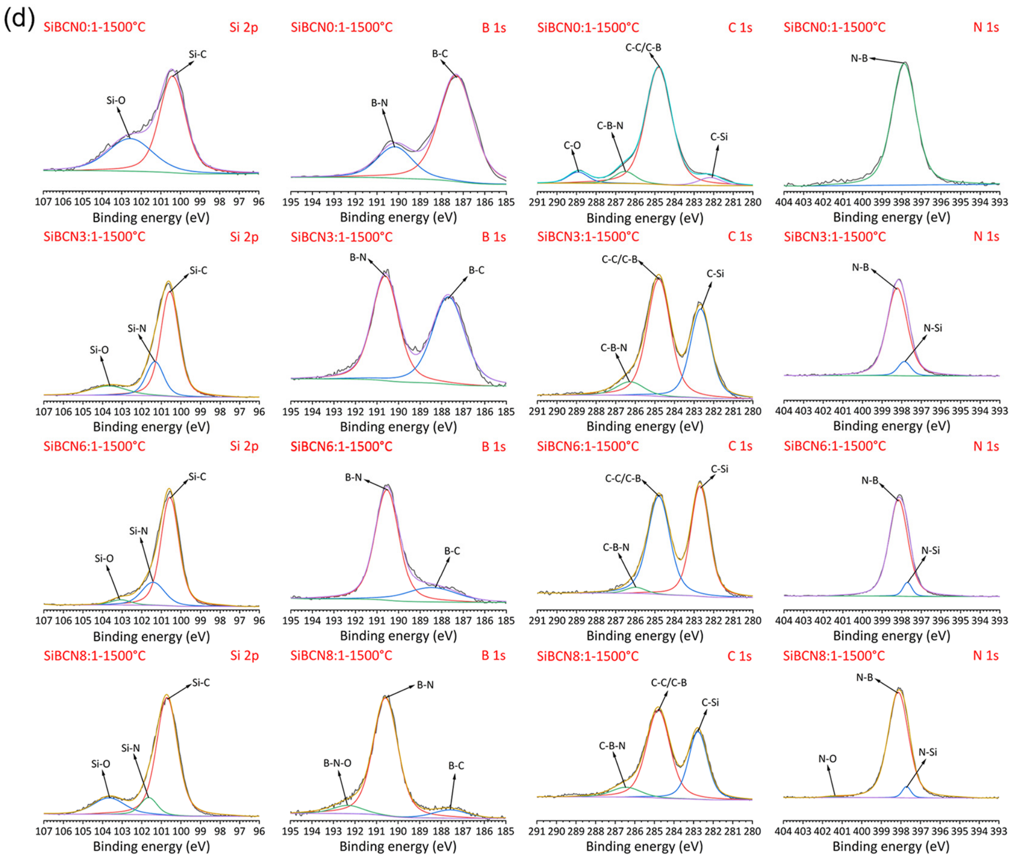

(d)
SiBCN0:1-1500°C
Si 2p
B 1s
C 1s
N 1s
SiBCN3:1-1500°C
SiBCN6:1-1500°C
SiBCN8:1-1500°C
Si-C
Si-O
Si-N
B-N
B-C
B-N-O
C-C/C-B
C-B-N
C-O
C-Si
N-B
N-Si
N-O
Binding energy (eV)

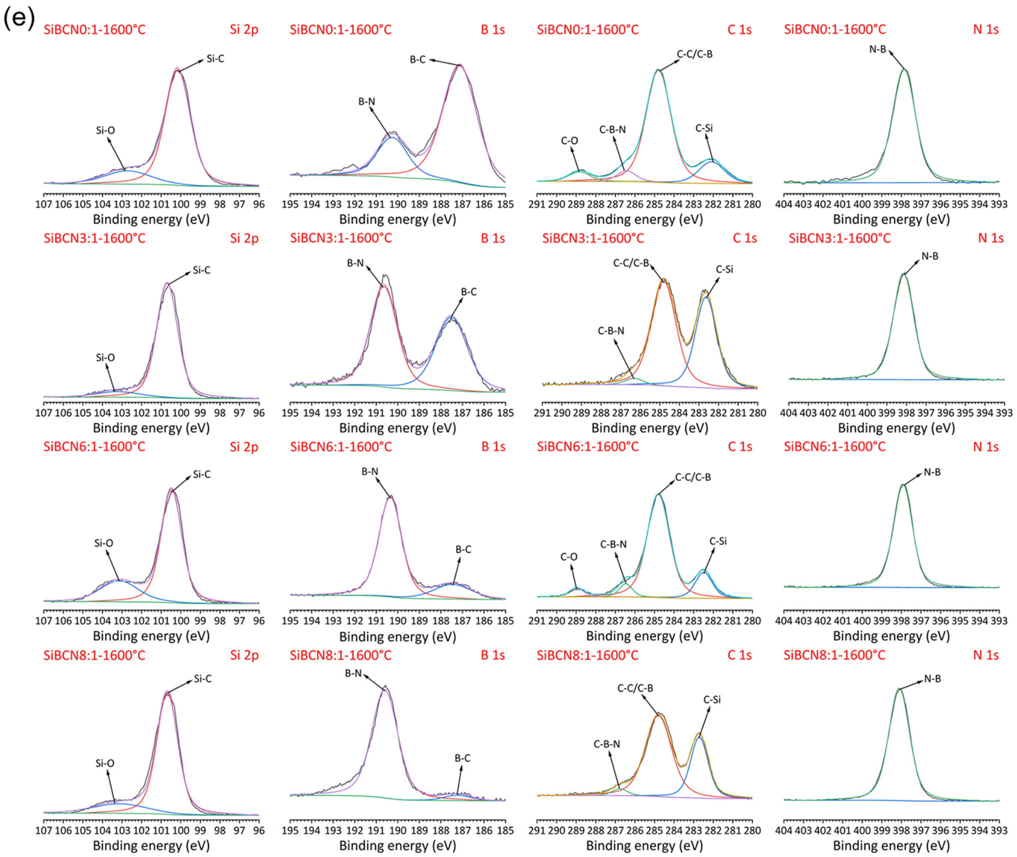


**Figure S2.** XPS spectra of SiBCN*x:y* pyrolyzed under Ar atmosphere at 1000 °C (**a**), 1200 °C (**b**), 1400 °C (**c**), 1500 °C (**d**), 1600 °C (**e**).

## 3. The surface topography of the ceramic bulk

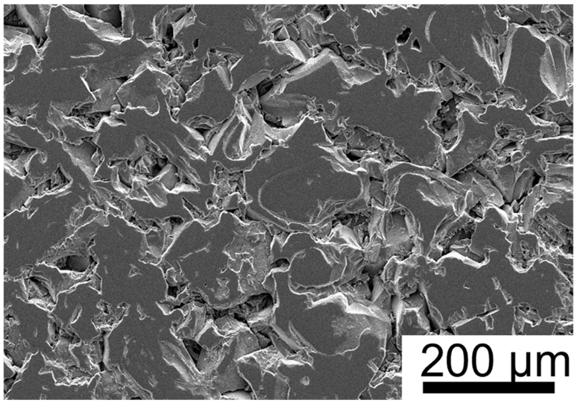


**Figure S3.** SEM image of the ceramic bulk.
